# Failed Roux-en-Y Gastric Bypass—Long-Term Results of Distalization with Total Alimentary Limb Length of 250 or 300 cm

**DOI:** 10.1007/s11695-022-06388-z

**Published:** 2022-12-02

**Authors:** Kamran Shah, Bent Johnny Nergård, Morten Wang Fagerland, Hjörtur Gislason

**Affiliations:** 1 Department of Surgery, Aleris Obesity Clinic, Aleris Hospital, Oslo, Norway; 2 Metabolic and Bariatric Unit, GB Obesitas, Skeppsbron 11, 211 20 Malmö, Sweden; 3 Oslo Centre for Biostatistics and Epidemiology, Research Support Services, Oslo University Hospital, Oslo, Norway

**Keywords:** Failed Roux-en-Y gastric bypass, Weight loss failure, Weight regain, Revisional surgery, Distalization, Total alimentary limb length, TALL, Common limb, Common channel, Malnutrition

## Abstract

**Background:**

Weight loss failure or weight regain may occur after Roux-en-Y gastric bypass (RYGB). Revisional surgery includes distalization. However, few studies have looked at the associations between the total alimentary limb length (TALL) and weight loss outcomes, none with long-term results.

**Objectives:**

Peri- and postoperative outcomes were assessed after employing TALL of either 250 cm or 300 cm in the failed RYGB.

**Methods:**

This study is a retrospective cohort analysis of 90 patients that underwent laparoscopic distalization between January 2006 and January 2016 due to failed RYBG. The index RYGB was modified to TALL of 250 cm (*n* = 48) or of 300 cm (*n* = 42) which entailed elongating the bilio-pancreatic limb (BPL) and transposing the Roux limb (RL) to a common limb (CL) of 100 cm and 150 cm, respectively. Long-term weight loss outcomes along with nutritional and vitamin status were analyzed.

**Results:**

Preoperative BMI at distalization was 38.6 kg/m^2^. After 8 years, excess weight loss (EWL) was 61.8%. No differences between the two groups were seen in weight loss outcomes or early surgical complication rates (6.7%). However, more vitamin and nutritional deficiencies were present in the TALL 250-cm group (50.0% and 35.4%, respectively) versus the TALL 300-cm group (33.3% and 14.3% respectively), which led to laparoscopic revision in 27 patients by lengthening the TALL with 100 cm. Patients with weight regain after index RYGB had in average 59.9% higher EWL than patients with EWL failure.

**Conclusion:**

Distalization of the failed RYGBP is safe and effective, but TALL should not be shorter than 300 cm (and CL 150 cm) due to high rates of malnutrition. Adequate supplementation and long-term follow-up are mandatory to prevent serious malnutrition.

**Graphical Abstract:**

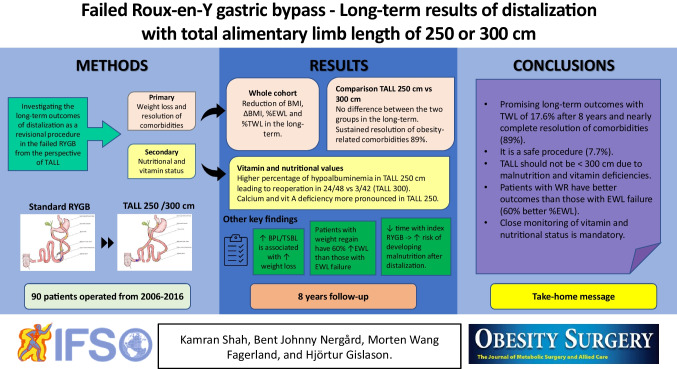

## Background

Roux-en-Y gastric bypass (RYGB) is an effective treatment option against obesity and its associated medical problems [1, 2]. However, with time, up to 40% of patients experience weight loss failure and/or weight regain, and different strategies have been implemented to counter this problem [3, 4]. Revisional surgery consists of pouch resizing, salvage banding [5], conversion to other operations [6] and distalization,  [7, 8] among others.

Due to its heterogeneous nature, distalization has varied surgical outcomes [9]. Few studies have looked at the outcomes of distalization from the perspective of reducing the total alimentary limb length (TALL) [10]. Previously, studies have shown excellent results of RYGB with TALL of 420 cm as a primary procedure in patients with BMI > 50 kg/m^211^ or reduction of TALL with a common limb (CL) length of 150 cm and 200 cm [11]. By tailoring the TALL to either 250 or 300 cm as a revisional procedure after failed RYGB due to weight regain and/or weight loss failure, we hypothesize that weight loss outcomes will improve in the long-term follow-up.

## Methods

This study is a retrospective analysis of prospectively collected data on patients with obesity who underwent revisional surgery due to weight loss failure and/or weight regain after a failed RYGB from January 2006 to January 2016 at a private high-volume bariatric center. The Reinhold criteria were used to define EWL failure (EWL after 18 months postoperatively of less than 50%) [12]. Weight regain was defined as gaining more than 15% of nadir weight achieved. When a combination of weight regain and excess weight loss failure were present, patients were categorized according to which of the two was the most predominant.

**Fig. 1:**
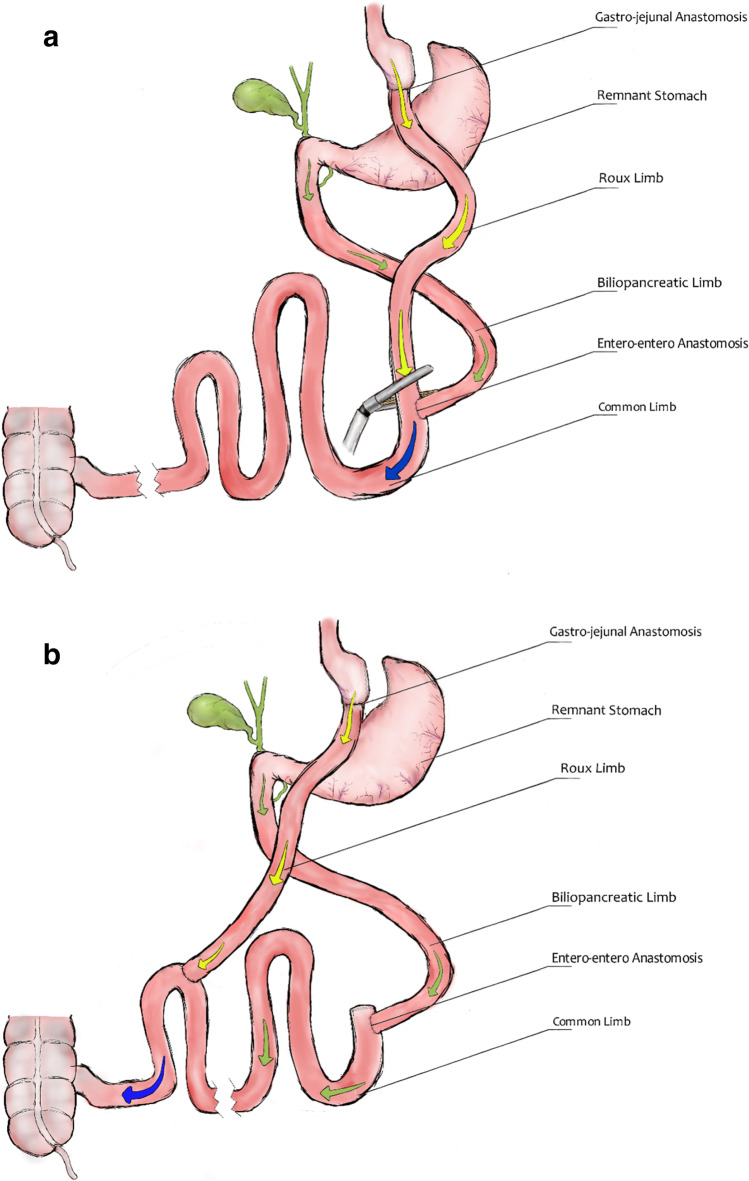
**a** Index RYGB before distalization. **b** Distalization of RYGB with shortened TALL

Patients were referred from third-party centers or came from our institution, and all underwent nutritional evaluation and counseling along with psychiatric appraisal if deemed necessary. Preoperative evaluation included upper gastrointestinal double contrast X-ray series and endoscopy to exclude fistulas and other anatomical abnormalities. Cases were discussed in multidisciplinary team conferences and decision for revisional surgery was made when no contraindications were present. The index RYGB in the vast majority of patients in Scandinavia is constructed with a 150-cm roux limb and a 60-cm BP limp which would approximate a TALL of 560 cm with a mean total small bowel limb length of 620 cm. From our previous studies, the type 2 distalization with a TALL of 420 cm in primary surgery had shown excellent results in those with a BMI above 50 kg/m^2^ [13]. Therefore, it was decided to initiate a pilot study where a TALL of 400 cm would be employed. Thus, in those deemed suitable, distalization was performed by dividing the RL at the entero-enterostomy and transposing it closer to the ileocecal valve thereby shortening the TALL (see Fig. 1a and b). The initial results were disappointing in terms of weight loss; thus, the TALL in the subsequent patients was shortened further to 250 cm with a common channel of 100 cm.

However, due to an increased prevalence of malnutrition, we decided in the subsequent patients to transpose the AL to 150 cm from the ileocecal valve instead, thereby creating a TALL of 300 cm. As a result hereof, two groups were created with a TALL of 250 cm and 300 cm respectively, whose peri- and postoperative outcomes were assessed. Standard follow-up included scheduled visits three times first year, two times the following 2 years and later on yearly visits. At every visit, weight loss results and resolution of associated medical problems were registered. Close follow-up of nutritional status was effectuated with routine lab tests at least twice yearly.

Standard daily oral vitamin and mineral supplementation were administered as follows: two multivitamin tablets, 1000 mg calcium citrate, vitamin D_3_ 160 μg (alternatively intramuscular injection with 300,000 IE, 1–2 mL every 3^rd^ month), one tablet TrioBe (0.5 mg cyanocobalamins, 0.8 mg folic acid, and 3 mg pyridoxine) or vitamin B12 injection 1 mg i.m. every 1–3 months and 100 mg iron for all patients unless ferritin was above 500 μg/L (alternatively ferriderisomaltose injection 1 g i.v. every 6–12 month.

Vitamin deficiencies were defined as severe if they were below the national reference values and occurred at least once with ongoing standard vitamin and oral supplementation. In those cases, additional supplementation (either orally or intravenously) was administered. If intractable severe malnutrition was present, percutaneous gastrostomy tube (PEG) for nutritional purposes was laparoscopically placed in the antrum of the remnant stomach.

Complications and remission of comorbidities were reported according to ASMBS guidelines [14].

### Statistical Methods

Statistical analyses were performed using SPSS for Windows, version 22.0, and StataSE version 15. Values were reported as mean ± standard deviation if not mentioned otherwise. Comparison between groups was made with one-way ANOVA (analysis of variance), *t*-test, or chi-square test. A *P*-value < 0.05 was considered statistically significant.

To assess the variation between each potential dependent variable and the binary outcomes, we performed a univariate analysis. If variables in outcomes had a *P*-value less than 0.2 on the univariate analysis, then a multivariate logistic regression model was applied, where variables were considered significant at a *P*-value less than 0.05.

## Results

**Fig. 2:**
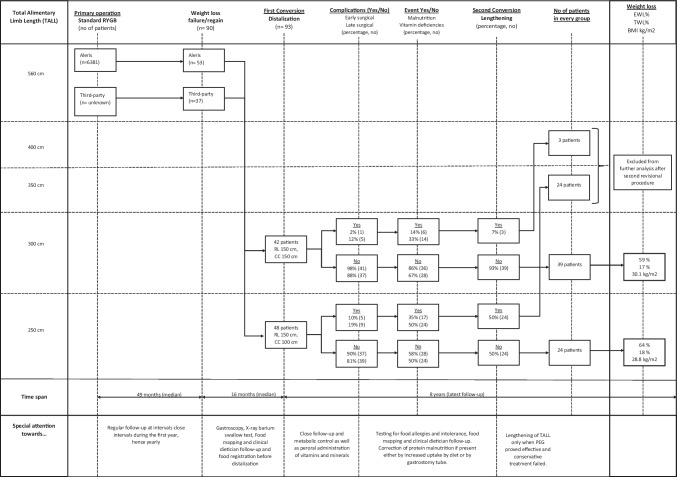
Flowchart depicting flow of patients in relation to TALL, time, and specific events

From January 2006 to January 2016, 90 patients (53 from our own institution and 37 referred from third-party hospitals) were converted to a distal RYGB at a median of 65 months (range 15–320 months) after the index RYGB (Fig. 2). Female/male ratio was 80:20 and mean age 44.9 ± 10.0 years at the time of revision. Mean BMI was 47.9 ± 6.7 kg/m^2 ^at index RYGB and 38.6 ± 5.8 kg/m^2^ at distalization (%EWL 41.1% and %TWL 19.1%). Forty-one patients underwent distalization due to weight regain while 49 patients had excess weight loss failure.

Initially, distalization was performed with the construction of a total alimentary limb length (TALL) of 250 cm (*n* = 48). Due to the development of severe malnutrition in several patients with time, we found it necessary to entail a TALL of 300 cm in the subsequent patients instead (*n* = 42). Measurements of limb lengths are shown in Table 1.

**Table 1:** Small bowel limb lengths in TALL 250 cm (RL 150 cm, CL 100 cm, *n* = 48) and TALL 300 cm (RL 150 cm, CL 150 cm, *n* = 42) (median values and range)

	BPL (cm)	TSBL (cm)	BPL/TSBL (%)
TALL 250 cm	358 (150–610)	610 (400–860)	58 (38–71)
TALL 300 cm	324 (170–510)	620 (470–810)	51 (36–63)

*TALL* total alimentary limb length, *RL* Roux limb, *CL* common limb, *BPL* bilio-pancreatic limb, *TSBL* total small bowel length

At 1-year follow-up after distalization, mean BMI had decreased to 29.4 ± 4.7 kg/m^2^ while %EWL and %TWL were 73.1 ± 35.4 and 23.0 ± 9.1, respectively. Long-term follow-up rate (up to 8 years) and weight loss results are listed in Table 2.

**Table 2:** Weight loss progression at follow-up and follow-up rate for the whole cohort

	BMI kg/m^2^	Δ BMI	%EWL	%TWL	Follow-up (%)
Index RYGB	47.9 ± 6.7	-	-	-	-
At distalization	38.6 ± 5.8	-	-	-	-
6 months	32.0 ± 5.2	6.6 ± 3.1	54.7 ± 28.4	16.9 ± 6.6	90/90 (100)
1 year	29.4 ± 4.7	9.1 ± 4.3	73.1 ± 35.4	23.0 ± 9.1	90/90 (100)
2 years	28.5 ± 4.1	9.9 ± 5.1	75.7 ± 31.8	24.5 ± 10.3	81/81 (100)
3 years	28.5 ± 4.1	9.9 ± 5.7	73.8 ± 32.3	24.7 ± 11.4	69/77 (89.6)
4 years	28.7 ± 4.3	9.3 ± 5.9	72.5 ± 40.4	23.0 ± 12.0	63/70 (90.0)
5 years	28.3 ± 3.3	9.1 ± 6.0	69.5 ± 35.0	23.0 ± 11.5	50/55 (90.9)
6 years	28.4 ± 3.3	8.2 ± 5.7	67.5 ± 37.0	21.0 ± 11.6	37/38 (97.3)
7 years	27.9 ± 3.6	6.3 ± 4.0	69.7 ± 40.7	19.7 ± 11.8	23/23 (100)
8 years	29.2 ± 2.4	4.9 ± 3.4	61.8 ± 41.2	17.3 ± 10.3	14/14 (100)

*RYGB* Roux-en-Y gastric bypass, *BMI* body mass index, *%EWL* percent excess weight loss, *%TWL* percent total weight loss

Specification of weight loss outcomes classified by TALL is provided in Table 3. Overall, higher rates of %EWL and %TWL was seen in the group with TALL 250 cm, although the difference between the groups did not reach statistical significance except for %EWL in the first year.

**Table 3:** Weight loss progression at follow-up differentiated on TALL 250 cm vs TALL 300 cm

		% EWL			%TWL	
	TALL 250 (*n* = 48)	TALL 300 (*n* = 42)	*P-*value	TALL 250 (*n* = 48)	TALL 300 (*n* = 42)	*P-*value
6 months	60.3 ± 32.9	48.2 ± 20.2	0.0418	17.0 ± 7.1	16.8 ± 5.8	0.8850
1 year	79.6 ± 39.8	64.5 ± 26.4	0.0396	23.8 ± 9.9	21.9 ± 7.9	0.3217
2 years	80.2 ± 33.3	69.8 ± 28.8	0.1424	25.5 ± 11.1	23.2 ± 8.9	0.5589
3 years	76.8 ± 34.1	69.0 ± 28.6	0.3324	25.3 ± 11.2	23.8 ± 11.8	0.5990
4 years	77.7 ± 42.6	63.3 ± 34.0	0.1765	24.0 ± 11.3	21.0 ± 12.9	0.3429
5 years	73.1 ± 29.0	62.9 ± 43.3	0.3290	24.5 ± 10.9	20.1 ± 12.0	0.2034
6 years	73.4 ± 28.2	55.0 ± 48.4	0.1522	22.5 ± 12.2	18.1 ± 9.9	0.2845
7 years	69.4 ± 28.8	70.4 ± 66.8	0.9608	20.3 ± 11.4	18.0 ± 12.4	0.7010
8 years	63.8 ± 17.1	58.9 ± 61.6	0.8217	17.8 ± 5.6	16.7 ± 7.3	0.7701

*TALL* total alimentary limb length, *%EWL* percent excess weight loss, *%TWL* percent total weight loss; mean value (standard deviation)

Linear regression showed that a longer BPL was associated with a higher %EWL (coefficient = 0.10, 95%CI 0.008–0.20; *P* = 0.034) and %TWL (coefficient = 0.032, 95%CI 0.005–0.058); *P* = 0.020). Thus, an increase in 100 cm in BPL was associated with an increase of 10% and 3.2% in EWL and TWL, respectively. Similarly, increasing the CL with 50 cm was associated with a 12.5% reduction in EWL (95% CI 0.8–24, *P* = 0.036) and a 3.5% reduction in TWL (95% CI 0.45–7.0; *P* = 0.026). Higher BPL/TSBL ratio was associated with higher rates of %EWL (coefficient = 1.28; 95%CI 0.13–2.43; *P* = 0.030) and %TWL (coefficient = 0.38; 95%CI 0.049–0.70; *P* = 0.025).

Postoperative BMI ( 95%CI − 1.19–3.20; *P* = 0.37), %EWL (95%CI − 26.6–8.06; *P* = 0.29) and %TWL (95% CI − 5.85–4.13; *P* = 0.74) after the index RYGB were not different between the patients with EWL failure versus weight regain. However, after the distalization, patients with weight regain had in average 59.9% higher EWL than patients with EWL failure (95% CI − 76.0 to − 43.7; *P* < 0.0001).

Prior to the index RYGB, 24.4% (22/90) had type 2 diabetes (T2D), 32.2% (29/90) had hypertension (HT), 26.7% (24/90) had dyslipidemia, and 5.6% (5/90) has obstructive sleep apnoea (OSA). 45.6% did not have any comorbidities. Prior to the distalization, 14.4% (13/90) had type 2 diabetes (T2D), 14.4% (13/90) had hypertension (HT), 7.8% (7/90) had dyslipidemia, and 3.3% (3/90) had obstructive sleep apnea (OSA). 71.1% (64/90) did not have any associated medical problems. After distalization, one patient had T2D and three patients had HT, while 95.6% did not have any associated medical problems (see Table 4). Multivariable logistic regression did not show any confounding effect on resolution of associated medical problems from CL, RL, BPL, TALL, TSBL, TSBL, WR, or EWL failure.

**Table 4:** Prevalence of comorbidities related to operations, no and percentage

	Prior to index RYGB	Prior to distalization	After distalization
DM2	22 (24.4%)	13 (14.4%)	1 (1.1%)
HT	29 (32.2%)	13 (14.4%)	3 (3.3%)
DL	24 (26.7%)	7 (7.8%)	0 (0%)
OSA	5 (5.6%)	3 (3.3%)	0 (0%)
No comorbidities	41 (45.6%)	64 (71.1%)	86 (95.6%)

*RYGB* Roux-en-Y gastric bypass, *DM2* diabetes mellitus type 2, *HT* hypertension, *DL* dyslipidemia, *OSA* obstructive sleep apnea

Logistic regression showed no association between gender, age, time elapsed from index RYGB to distalization, comorbidities, TALL, or BPL/total small bowel length (TSBL) ratio on who experienced weight regain or excess weight loss failure (see Table 5). Cox regression showed that an increase of BMI at primary operation with one point led to a reduction in risk of weight regain with a factor 0.91 (95%CI 0.87–0.97; *P* = 0.001). Linear regression analysis showed a positive relationship between time elapsed from index RYGB to distalization and incidence of malnutrition with a *P*-value of 0.02 (coefficient =  − 0.003, 95% CI − 0.0049–0.00044 *P* = 0.020).

**Table 5:** Factors associated with weight regain vs excess weight loss failure. Results from logistic regression (odds ratio) and Cox regression (hazard ratio)

	Odds ratio (95% CI); *P*-value	Hazard ratio (95% CI); *P*-value
Male vs female	0.87 (0.18 to 4.13); *P* = 0.86	0.96 (0.29 to 3.12); *P* = 0.94
Age	0.99 (0.95 to 1.03); *P* = 0.54	0.97 (0.94 to 1.01); *P* = 0.10
Time elapsed	1.00 (0.99 to 1.01); *P* = 0.50	*Not computable*
BMI primary operation	0.95 (0.88 to 1.01); *P* = 0.085	0.91 (0.87 to 0.97); *P* = 0.001
Comorbidities before primary op	1.18 (0.51 to 2.72); *P* = 0.71	1.17 (0.63 to 2.19); *P* = 0.61
Total alimentary limb length	1.00 (0.996 to 1.005); *P* = 0.68	1.00 (0.995 to 1.002); *P* = 0.62
BPL/TSBL after index	0.95 (0.72 to 1.25); *P* = 0.69	1.07 (0.86 to 1.33); *P* = 0.54

*BPL* bilio-pancreatic limb, *TSBL* total small bowel limb length

### Complications

Total early surgical (minor and major) complication rate was 6.7%. One patient experienced early postoperative bleeding necessitating re-laparoscopy and five patients underwent laparoscopic revision due to small bowel obstructions at the entero-entero anastomosis. No leaks were seen. Overall, surgical reoperation rate was 16.7%. Among them are four negative laparoscopies and five symptomatic internal hernia. Malnutrition was present in 25.5% (*n* = 23) of the total cohort while 42.2% (*n* = 38) of the patients had vitamin deficiencies (see Table 6).

**Table 6:** Complications, no of patients

	Early surgical complications		Late surgical complications		Vitamin deficiencies	Malnutrition
	Minor	Major	Minor	Major		
TALL 250	0	5	5	3	24	17
TALL 300	0	1	4	2	14	6
*P*-value	NA	.1273	.1273	.7585	.1103	.0219

Definition of minor and major complications according to ASMBS criteria

*TALL* total alimentary limb length

Dichotomizing the cohort into TALL 250 cm and TALL 300 cm shows no statistically significant differences between the groups in early or late surgical complications. However, higher incidence of vitamin deficiencies and malnutrition were noted in the TALL 250-cm group (50.0% and 35.4%., respectively) versus the TALL 300-cm group (33.3% and 14.3% respectively).

There was no mortality related to the surgery.

### Vitamin and Nutritional Status

After distalization, the albumin and hemoglobin levels were significantly lower in both groups as compared to pre-distalization. The PTH values were also higher in the TALL 250-cm group as compared to the TALL 300-cm group (Table 7). The number of patients with malnutrition defined as albumin levels below the normal reference value) is significantly higher in the TALL 250-cm group (40.5%) versus both the TALL 300-cm group (14.3%) and the pre-distalization group (15.6%) (see Table 8). Despite supplementation, a significantly higher number of patients had calcium deficiencies in both TALL groups after distalization, while vitamin A deficiencies are only noted in the TALL 250-cm group (Table 8).

**Table 7:** Mean nutritional lab and vitamin levels at different times with differentiation between TALL 250 cm and TALL 300 cm at 5-year follow-up after distalization

	All patients		TALL 250 cm	TALL 300 cm	
	Before index RYGB (*n* = 74)	Before distalization (*n* = 90)	After distalization (*n* = 48)	After distalization (*n* = 42)	*P*-value
Albumin (g/L)	42.7 ± 2.3	41.0 ± 2.0	35.8 ± 5.2	38.5 ± 6.1	.0259
Hemoglobin (g/DL)	14.0 ± 1.4	13.2 ± 0.9	11.9 ± 1.2	12.4 ± 1.3	.0061
Iron (μmol/L)	11.6 ± 3.4	17.4 ± 9.1	14.0 ± 4.9	13.8 ± 5.1	.8501
Ferritin, μg/L	44.3 ± 13.7	44.1 ± 37.9	167.2 ± 202.2	100.7 ± 101.9	.0572
Calcium (mmol/L)	2.32 ± 0.1	2.27 ± 0.1	2.18 ± 0.16	2.20 ± 0.12	.5090
PTH (pmol/L)	NA	NA	9.7 ± 4.5	7.9 ± 2.8	.0276
Cobalamin (pmol/L)	292.7 ± 38.6	430.2 ± 294.4	668.8 ± 416.2	693.2 ± 416.9	.7822
Vitamin A (μmol(L)	NA	NA	1.29 ± 1.0	1.37 ± 0.72	.6683
Vitamin D (nmol/L)	48.0 ± 21.8	41.0 ± 2.0	73.0 ± 33.6	78.6 ± 28.1	.3973
Vitamin E (μmol/L)	NA	NA	15.4 ± 5.5	16.9 ± 6.3	.2310
Vitamin K	NA	NA	0.061 ± 0.129	0.043 ± 0.071	.4237
Se-Zinc (μmol/L)	15.0 ± 1.25	13.1 ± 1.7	10.9 ± 2.2	11.1 ± 1.8	.6411

*TALL* total alimentary limb length

*Unpaired t test, significance level .05*

**Table 8:** Number and percentage of patients with vitamin and nutritional values below normal range in spite of conventional treatment (oral and intravenously)

		All patients (*n* = 90)		TALL 250 (*n* = 48)	TALL 300 (*n* = 42)
	Reference values	Before index RYGB (*n* = 74)	Before distalization (*n* = 90)	After distalization	
Albumin	36–48 g/L	1 (1.4%)	14 (15.6%)**^a^	17 (40.5%)**^b^	6 (14.3%)**^c^
Hemoglobin	g/DL	9 (12.2%)	29 (32.2%)**^a^	22 (45.8%)	15 (35.7%)
Iron	9–34 μmol/L	14 (18.9%)	14 (15.5%)	5 (10.4%)	9 (21.4%)
Ferritin	μg/L	24 (32.4%)	4 (4.4%)***^a^	2 (4.2%)	1 (2.4%)
Calcium	2.15–2.51 mmol/L	5 (6.8%)	5 (5.6%)	15 (38.1%)***^b^	11 (26.2%)**^b^
Cobalamin	> 275 pmol/L	24 (32.4%)	7 (7.8%)***^a^	5 (10.4%)	4 (9.5%)
Vitamin A	1.2–3–3 μmol/L	NA	23 (25.6%)	25 (52.1%)**^b^	14 (33.3%)
Vitamin D	> 50 μmol/L	19 (25.7%)	11 (12.2%)*^a^	10 (20.8%)	7 (16.7%)
Vitamin E	13–40 μmol/L	N	15 (16.7%)	10 (20.8%)	10 (23.8%)
Se-zinc	10–17 μmol/L	0 (0%)	19 (21.1%)	14 (29.2%)	10 (23.8%)

Chi-square test, significance level .05

^a^Compared to “Before index RYGB”

^b^Compared to “Before distalization”

^c^Compared to “TALL 250”

^*^P < 0.05

^**^P < 0.01

^***^P < 0.0001

Both the ratio of BPL/TSBL and CL were associated with nutritional deficiencies. Thus, those with nutritional deficiencies had in average 3.87% higher BPL/TSBL ratio as compared to those without deficiencies (− 7.16 to − 0.57; *P* = 0.022). Likewise, those without nutritional deficiencies had in average 33.9 cm longer CL compared to those with deficiencies (− 22.7 to 45.1; *P* < 0.0001).

Diarrhea was noted as occurring periodically in 10.4% (5/48) in the TALL 250-cm group vs 4.8% (2/42) in the TALL 300-cm group. Periodically, diarrhea was treated successfully with conservative treatment that included behavioral changes, nutritional counseling, and pharmacotherapy.

Three patients with TALL 300 cm and 24 patients with TALL 250 cm had severe intractable malnutrition, hypoproteinemia, and diarrhea refractory to medical treatment. These patients underwent another revisional surgery where the CL was lengthened with 100 cm at the expense of the BPL thus ending with a TALL of 400 cm and 350 cm, respectively. The postoperative course in this subgroup of patients was uneventful except for one patient who was re-operated due to an obstruction at the entero-entero anastomosis and one patient that unfortunately died 4 days postoperatively due to sepsis caused by a leakage. All issues of malnutrition, hypoproteinemia, and diarrhea were resolved successfully in the remainder patients. After the second revisional surgery, this subgroup of patients were excluded from the total cohort and further statistical analysis in the two groups.

## Discussion

### Weight Loss Outcomes and Follow-up

This study demonstrated the efficacy of distalization in the failed RYGB with promising mid-term (3 years) and long-term (up to 8 years) weight loss results with %TWL of 24.7% and 17.2%, respectively. Although similar mid-term data have been reported in several studies [4, 10], [15], [16], we are not aware of other publications with long-term outcomes. It seems plausible to hypothesize that the initial mid-term weight loss achieved after the distalization would be ameliorated to some degree in long term as weight regain is likely to take place. Therefore, long-term data are of crucial importance when evaluating the effect of distalization. Furthermore, this study is also distinctive due to the large patient cohort (90 patients) included and high follow-up percentage (100%). To our knowledge, the only other study with a similar large patient cohort is the study of Ghiassi [10], where 96 patients were included but most other studies are hampered by a small number of patients.

In a previous study, we showed the superiority of a distal gastric bypass as a primary procedure in the patient with severe obesity by constructing a TALL of 420 cm^11^. We did not deploy the same length of TALL in this study as an initial pilot study (case series) showed disappointing weight loss results when constructing a TALL of 400 cm in the failed RYGB (non-published results). We therefore hypothesized that in order to achieve acceptable long-term weight loss results, a TALL well below 400 cm would be necessary. Therefore, in this study, a TALL of 250 cm was created in the failed RYGBP. However, our initial experience with a high incidence of malnutrition in the TALL 250 cm patients made us lengthen the TALL to 300 cm instead. Both operations had similar effects on weight loss, but in the absence of statistically significant differences between the two groups, we cannot say that weight loss outcomes were more superior in the TALL 250-cm group versus the TALL 300-cm group.

When comparing the outcomes of the present study with the results of Ghiassi et al., they started by performing distalization of the failed RYGBP with a TALL of 250–300 cm and after 11 patients lengthened the TALL to 400–450 cm due to high incidences of malnutrition. They reported 1-year weight loss outcomes in a cohort of 42 of 60 patients (70% follow-up) with a TALL of 400–450 cm as follows: EWL 41.9%, TWL 15.3%, and reduction of 6.4 BMI units. Our corresponding 1-year weight loss outcomes in the TALL 300-cm group (with 42 patients) were as follows: EWL 64.5%, TWL 21.9%, and reduction of BMI 9.1 units. After 2 and 3 years, follow-up dropped to 50% in the study of Ghiassi et al. and is not comparable to our study because weight loss outcomes usually become less favorable when follow-up is better. In a long-term setting, one would hypothesize that a TALL of 400–450 cm would ameliorate the initial achieved weight loss to some extent or perhaps even completely.

The lesson learned from both studies is that a construction of TALL 250 cm is too much. In our setting with a meticulous and high follow-up with a tight metabolic surveillance, a TALL of 300 cm seems to be safe. The number of patients undergoing the second revisional procedure in the TALL 300-cm group was 3/42 and while a TALL of 350 cm has not been explored in this study, one would expect that this length would reduce or perhaps diminish the necessity of a second revisional procedure due to malnutrition. As such, it could serve as preliminary foundation for recommending a TALL of 350 cm while awaiting further studies to see how weight loss outcomes would be affected. However, in countries where follow-up is more challenging, a TALL of 300 cm might even be too short. Perhaps the ideal length of TALL in distalizing the failed RYGBP would be 350 cm when we consider weight loss outcomes as well as the malnutrition issues that those who underwent the second revisional procedure encountered.

### Safety, Resolution of Obesity-Related Comorbidities, and Overall Reoperation Rate

Another key finding in this study include the safety of both TALL 250 and 300 cm without differences in peri- or postoperative morbidity and mortality along with high rates of resolution (96.5%) of obesity-related comorbidities.

The overall reoperation rate of 16.7% (15/90 patients) encompasses both early reoperations (within the first 30 days) and late reoperations (follow-up of more than 8 years). Six patients (6/90) were included in the first category as one patient underwent re-laparoscopy due to bleeding while five patients had small bowel obstructions. This is similar to the study of Ghiassi wherein the early surgical reoperation rate was 5.2% (5/96). In the latter category (late reoperations), five patients underwent a reoperation because of internal hernia, while in four patients the laparoscopy was negative. A low-threshold strategy for laparoscopy has been implemented since the early start in our team in order to maximize patient safety and not miss potential positive cases. Omitting the negative laparoscopies gives a total reoperation rate of 12.2%. These relatively high percentages reflect the meticulous follow-up in the long-term but are still relatively low compared to reoperation rates in other revisional surgeries [4, 10], [17].

### Malnutrition and Vitamin Deficiencies

High rates of malnutrition and vitamin deficiencies accompanied the TALL 250-cm group. This finding is consistent with the study of Ghiassi [10], wherein a TALL of 400–450 cm at 3-year follow-up had fewer nutritional deficiencies than a TALL of 250–300 cm but where the effect on calcium, parathyroid hormone, and vitamins A and D was still an issue of concern in both groups. However, the Ghiassi study was characterized by a small number of patients available for follow-up and only extended to a mid-term follow-up.

The construction of TALL 250 cm was also tested in the failed RYGB due to weight regain in 30 patients by Felsenreich et al. [15] with a follow-up of 1 year. Thirty percent of the patients underwent a second revisional procedure due to malnutrition with a lengthening of TALL to 400 cm.

Our study confirms, as Sugarman [18] and other more recent publications [7, 15] have demonstrated, that a short common limb is associated with more nutritional and vitamin deficiencies. Furthermore, our study confirms that the site of construction of the entero-entero anastomosis should be at least 150 cm from the ileocecal vale.

### Other Key Findings

We also found that a higher BPL/TSBL ratio was associated with higher rates of %EWL and %TWL. This supports the notion that a longer total small bowel length must be accompanied by a correspondingly longer BPL for better weight loss results. Tailoring fixed ratios of BPL lengths without accounting for the total small bowel limb length may lead to suboptimal weight loss results. We have not encountered other studies that have investigated this association in revisional surgery.

We also found a positive relationship between time elapsed from index RYGB to revisional surgery and development of malnutrition after revisional surgery. This indicates that the longer a patient has the index RYGB, the less likely he/she is to develop malnutrition after the revisional procedure. From other studies, we know that after the RYGB several small bowel changes take place, such as increased intestinal permeability, intestinal adaption, and mucosal hyperplasia [19–21]. Whether this association is caused by these small bowel changes or whether other explanations exist are yet to be explored in upcoming studies. Nevertheless, it seems plausible that the longer the patient is exposed to these small bowel adaptions, the less likely malnutrition takes place after a distal gastric bypass.

It also seems that the patients that benefit the most from distalization in terms of EWL are the subpopulation that initially had a good weight response, but regained weight as opposed to those who had excess weight loss failure after the index RYGB. We are not aware of previous studies that has looked at this association in revisional surgery. The underlying reasons for weight regain are believed to be secondary to behavioral changes, hormonal differences in gut peptides, or dietary adaptation to hypoglycemic episodes [22], and are thus different from the reasons to EWL failure. Therefore, it is plausible to believe that if EWL failure is the primary reason for a failed RYGB in the absence of anatomical abnormalities, re-doing the RYGB with a distalization may not counteract the underlying reasons for the initial weight loss failure as opposed to weight regain.

This should be taken into consideration when patients present for distalization so mutual expectations from the surgeon and the patient can be met.

Several studies have been published dealing with the issue of TALL in primary RYGB surgery. More focus with high-quality studies should be directed at the failed RYGB as revisional surgery is on the rise and have reached 15.4% of all bariatric surgery in 2018 according to the latest figures from ASMBS [23].

### Limitations

One of the major limitations of this study is the nature of the retrospective design study. Also, a minor sampling bias may be presented based on the availability of the patients. However, we deemed this negligible as our follow-up rate was 90% or above throughout the follow-up period of 8 years.

## Conclusion

Distalization of a failed RYGB due to weight loss failure is safe and effective but TALL should not be reduced to more than 300 cm (and common limb 150 cm) cm due to protein-calorie malnutrition and higher rates of nutritional and vitamin deficiencies. Adequate supplementation and long-term follow-up are mandatory to prevent serious malnutrition.

Our data does not support a fixed ratio of BPL/TSBL as a longer TSBL may lead to suboptimal weight loss outcomes in revisional surgery. Careful selection of subjects should be undertaken so that expectations from both the patient and the surgeon are met, as weight loss outcomes are not as convincing as when the failed RYGB is due to EWL failure as opposed to weight regain.

## Data Availability

All data generated or analyzed during this study are included in this article. Further inquiries can be directed to the corresponding author.
